# Myeloid But Not Endothelial Expression of the CB2 Receptor Promotes Atherogenesis in the Context of Elevated Levels of the Endocannabinoid 2-Arachidonoylglycerol

**DOI:** 10.1007/s12265-022-10323-z

**Published:** 2022-09-30

**Authors:** Elina Avraamidou, Moritz Nöthel, Melina Danisch, Laura Bindila, Susanne V. Schmidt, Beat Lutz, Georg Nickenig, Julian Jehle

**Affiliations:** 1 Department of Internal Medicine II Cardiology, Pneumology, Angiology, University Hospital Bonn, Venusberg-Campus 1, Building 13, 53127 Bonn, Germany; 2 Institute of Physiological Chemistry, University Medical Center of the Johannes Gutenberg University Mainz, Duesbergweg 6, 55128 Mainz, Germany; 3 Institute of Innate Immunity, Medical Faculty, University of Bonn, 53127 Bonn, NRW Germany

**Keywords:** Cannabinoid receptor type 2, Atherosclerosis, Reactive oxygen species, Endocannabinoid system, 2-Arachidonoylglycerol, JZL184

## Abstract

The endocannabinoid 2-arachidonoylglycerol (2-AG) is an inflammatory mediator and ligand for the cannabinoid receptors CB1 and CB2. We investigated the atherogenic mechanisms set in motion by 2-AG. Therefore, we created two atherosclerotic mouse models with distinct cell-specific knockouts of the CB2 receptor on either myeloid or endothelial cells. These mice were treated with JZL184, resulting in elevated plasma levels of 2-AG. After a high-fat high-cholesterol diet, atherosclerotic plaques were analyzed. The atherogenic effect of 2-AG was abrogated in mice lacking myeloid expression of the CB2 receptor but not in mice lacking endothelial expression of the CB2 receptor. In vitro, treatment of human monocytes with 2-AG led to the increased production of reactive oxygen species (ROS) and IL-1β. In conclusion, 2-AG shows an atherogenic effect in vivo, dependent on the presence of the CB2 receptor on myeloid cells. In addition, our in vitro data revealed 2-AG to promote inflammatory signalling in monocytes.

**Graphical abstract:**

2-Arachidonoylglycerol shows an atherogenic effect that is abrogated in mice lacking myeloid expression of the CB2 receptor.

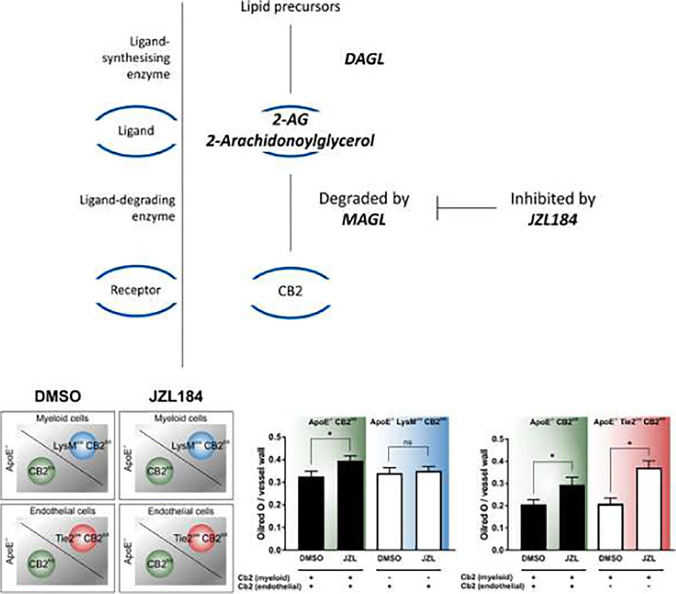

**Supplementary Information:**

The online version contains supplementary material available at 10.1007/s12265-022-10323-z.

## Introduction

The endocannabinoid 2-arachidonoylglycerol (2-AG) is a mediator of inflammation and ligand to the cannabinoid receptors CB1 and CB2, which are expressed on peripheral myeloid and human vascular endothelial cells [3, 31]. The CB2 receptor has traditionally been considered to exert anti-inflammatory and atheroprotective effects, such as decreasing macrophage accumulation in lesions, decreasing smooth muscle cell content in lesions, and decreasing apoptosis [28, 31, 32]. However, some conflicting data suggest that 2-AG can actually promote atherosclerosis and that the CB2 receptor may initiate inflammation as well as monocyte chemotaxis, infiltration, and activation under certain experimental conditions [5, 7, 9, 12, 22, 24, 30, 40]. Multiple molecular pathways that promote atherogenesis have been discovered to date, yet the molecular effects of 2-AG in atherogenesis still need further exploration. Such molecular mechanisms include the generation of reactive oxygen species, the secretion of myeloperoxidase, and the activation of the IL-6/IL-1β/CRP axis [2, 6, 14, 16, 26, 27, 33, 35, 36, 39].

In the present study, we investigated the role of the CB2 receptor on myeloid and vascular endothelial cells in promoting atherosclerosis under elevated 2-AG levels. This was investigated by using two cell-specific CB2 knockout mouse models. Both mouse models had an atherogenic background (*ApoE*^*−/−*^) and, additionally, distinct cell-specific knockouts of the CB2 receptor on either myeloid (*ApoE*^*−/−*^*LysM*^*cre*^*CB2*^*fl/fl*^) or endothelial (*ApoE*^*−/−*^*Tie2*^*cre*^*CB2*^*fl/fl*^) cells. Mice were treated with intraperitoneal injections of JZL184, an inhibitor of the 2-AG-degrading enzyme monoacylglycerol lipase (MAGL), which led to elevated 2-AG levels. Meanwhile, they were fed a high-fat and high-cholesterol diet for 4 weeks. The volume and composition of the plaques formed were analyzed. In vitro, human monocytes were stimulated with 2-AG and a CB2 receptor antagonist. We investigated the production of reactive oxygen and nitrogen species (ROS, RNS), the transcription of NADPH oxidases (NOX), the release of myeloperoxidase (MPO), and the production of IL-1β under stimulatory conditions.

## Materials and Methods

A detailed materials and methods section including a graphical summary of the experimental plan is available as an online supplement. In brief, two mouse models with an atherogenic background (*ApoE*^−/−^; *C57BL/6 J* genetic background; Charles River, Wilmington, USA) and distinct cell-specific knockouts of the CB2 receptor (CB2 gene) on either myeloid (*ApoE*^−/−^*LysM*^cre^*CB2*^fl/fl^) or endothelial (*ApoE*^−/−^*Tie2*^cre^*CB2*^fl/fl^) cells were created using the cre-lox system. Littermates without cell-specific cre-recombinase activity, and thus without any CB2 knockout (*ApoE*^−/−^*CB2*^fl/fl^), served as the control groups for both knockout strains. For each strain, the mice were divided into two groups, one of which was treated with 5 mg/kg body weight i.p. of the MAGL inhibitor JZL184 (Selleckchem, Munich, Germany), while the other group received the same volume of the vehicle PBS/Kolliphor/DMSO (AppliChem, Darmstadt, Germany) for 4 weeks. We chose a short treatment period of 4 weeks because we expected an increased plaque burden after treatment with JZL184. This protocol was established and validated in an earlier trial [12]. Approval was granted by the responsible German authority, the North Rhine Westphalian State Agency for Nature, Environment and Consumer Protection (reference number 84–02.04.2014.A419).

After 4 weeks of a high-fat (21% (w/w)) and high-cholesterol (1.25% (w/w)) diet S0279-S011 (Ssniff, Soest, Germany), mice were sacrificed, and the atheosclerotic plaques were analyzed by using Oil Red O (Sigma-Aldrich, St. Louis, USA), CD68 (α-CD68 rat-IgG2a antibody; Acris antibodies GmbH, Herford, Germany), and Ly6G (Ly6G purified clone 1A8, BD551459; Becton, Dickinson and Company, Franklin Lakes, USA) stainings. Ex vivo, ROS production was measured using the L-012 method (Berthhold Technologies GmbH, Germany), and plasma levels of IL-6 and IL-1β were quantified using a ProcartaPlex assay (PPX-02-MX323DE; Thermo Fisher Scientific, Vienna, Austria). In vitro, human monocytes were treated with 2-AG (Tocris Bioscience, Bristol, England), and the production of reactive oxygen species (ROS) and reactive nitrogen species (RNS) was studied using a fluorescent assay kit (ab139476; abcam, Cambridge, UK). Production of IL-1β (R&D Systems, Minneapolis, USA) and MPO (ab195212; abcam, Cambridge, UK) was measured by ELISA. Concentrations of MMPs 1 and 9 were quantified by R-Plex (Meso Scale Diagnostics, Rockville, Maryland, USA). Transcription of NOX1, NOX2 (CYBA, CYBB subunits), NOX4, and NOX5 was quantified by qPCR (Thermo Fischer Scientific Inc., Massachusetts, USA). Details of the TaqMan probes used in this study are listed in Supplementary Table S1.

Data are presented as the mean ± SEM. Data were analyzed using Microsoft Excel (Microsoft, Redmond, USA) and GraphPad Prism software (GraphPad Software, San Diego, USA). For the comparison of continuous and normally distributed variables between two groups, an unpaired Student’s two-sided *t* test was applied. For the comparison of three or more groups and normally distributed variables, a one-way ANOVA and subsequent Bonferroni correction were performed. In case of lacking Gaussian distribution, Kolmogorov–Smirnov test was applied for the comparison of two groups, and Kruskal–Wallis test with subsequent Dunn’s multiple comparisons for nonparametric testing was performed for three or more groups. *p* values < 0.05 were considered statistically significant.

## Results

In vivo, the impact of elevated 2-AG levels on atherogenesis was assessed in ApoE-deficient-mice (*ApoE*^*−/−*^) combined with a distinct cell-specific knockout of the CB2 receptor on either myeloid (*ApoE*^*−/−*^*LysM*^*cre*^*CB2*^*fl/fl*^) or endothelial cells (*ApoE*^*−/−*^*Tie2*^*cre*^*CB2*^*fl/fl*^). The effects of 2-AG in vitro were examined in human monocytes.

### Pharmacological Inhibition of MAGL Increases 2-AG Levels in Both Myeloid and Endothelial CB2 Receptor Knockout Models

Two different mouse models with cell-specific knockouts of the CB2 receptor in either myeloid cells (*ApoE*^*−/−*^*LysM*^*cre*^*CB2*^*fl/fl*^) or endothelial cells (*ApoE*^*−/−*^*Tie2*^*cre*^*CB2*^*fl/fl*^) were treated with the MAGL inhibitor JZL184 or vehicle for 4 weeks. Cre-negative littermates (*ApoE*^*−/−*^*LysM*^*wt*^*CB2*^*fl/fl*^ and *ApoE*^*−/−*^*Tie2*^*wt*^*CB2*^*fl/fl*^) served as controls. All animals were fed a high-fat and high-cholesterol diet for the duration of the treatment.

As anticipated, inhibition of MAGL by JZL184 led to a significant increase in the plasma levels of 2-AG compared to the vehicle-treated control animals:

For mice lacking the myeloid CB2 receptor (*ApoE*^*−/−*^*LysM*^*cre*^*CB2*^*fl/fl*^), the concentrations of 2-AG in the plasma were 59.4 ± 7.0 pmol/ml (JZL184 treatment) vs. 16.9 ± 2.2 pmol/ml (with DMSO; *n* = 16–17; *p* < 0.0001). This increase was also observed in the wildtype control group, where 2-AG yielded 58.0 ± 5.2 pmol/ml (JZL184 treatment) and 18.5 ± 3.0 pmol/ml (with DMSO; *n* = 15–16; *p* < 0.0001) (Supplementary Table S2).

Congruent results were seen in mice lacking the endothelial CB2 receptor (*ApoE*^*−/−*^*Tie2*^*cre*^*CB2*^*fl/fl*^) as well as in their cre-negative littermates (*ApoE*^*−/−*^*Tie2*^*wt*^*CB2*^*fl/fl*^) after pharmacological inhibition of MAGL with JZL184. The plasma concentrations for the CB2 knockout mice were 73.1 ± 6.4 pmol/ml (JZL184 treatment) vs. 33.5 ± 2.9 pmol/ml (with DMSO; *n* = 13–14; *p* < 0.0001). The control animals displayed an equivalent increase in 2-AG concentrations (71.4 ± 5.1 pmol/ml after JZL184 treatment vs. 42.7 ± 3.3 pmol/ml with DMSO; *n* = 14–17; *p* < 0.0001). Similar effects were seen in the aortic tissue of *ApoE*^*−/−*^*Tie2*^*cre*^*CB2*^*fl/fl*^ animals (239.5 ± 36.2 pmol/ml, JZL184 vs. 61.0 ± 14.1 pmol/ml, DMSO; *n* = 13–14; *p* = 0.0001), just as was seen in their cre-negative littermates (173.2 ± 20.0 pmol/ml, JZL184 vs. 68.9 ± 13.2 pmol/ml, DMSO; *n* = 15–17; *p* = 0.0002) (Supplementary Table S2).

Meanwhile, the plasma concentrations of arachidonic acid, as well as palmitoylethanolamide, were unaffected by JZL184 treatment (Supplementary Table S3).

### Blood Pressure, Heart Rate, and Body Weight

Several clinical parameters, blood pressure, heart rate, and body weight, were acquired for mice of all genotypes and treatments. None of them, neither for the myeloid CB2 knockouts, the endothelial CB2 knockouts, nor the cre-negative animals, was affected by JZL184 treatment, as detailed in Supplementary Table S4.

### 2-AG-Induced Atherogenesis in ApoE-Deficient Mice Is Abrogated in Mice Lacking Myeloid CB2 Receptor Expression

**Fig. 1:**
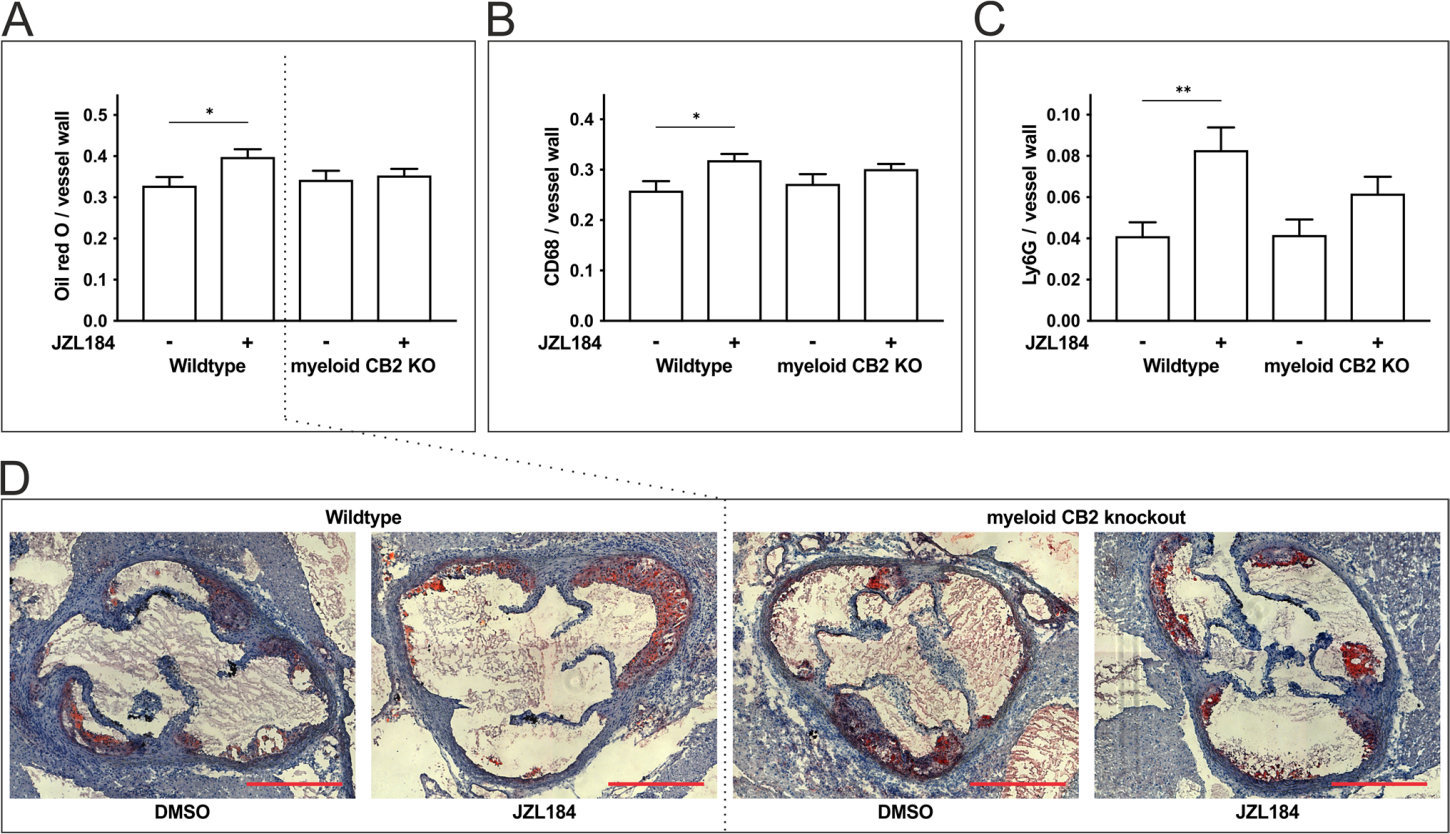
Assessment of atherogenic effects in mice lacking the myeloid CB2 receptor and in wildtype animals after treatment with JZL184. Staining of aortic valve sections in mice with a myeloid-specific knockout of the CB2 receptor and the respective wildtype controls. **a, d** Atherosclerotic plaque volume (Oil Red), **b** monocyte/macrophage (CD68), and **c** neutrophil (Ly6G) extravasation in mice with and without myeloid CB2 receptor expression. Data are presented as the mean ± standard error of the mean; *n* = 12–17; * ≤ 0.05, ** *p* ≤ 0.01, as assessed by Student’s *t* test. Scale bar, 500 μm. DMSO, dimethyl sulfoxide; JZL184, inhibitor of monoacylglycerol lipase

The atherosclerotic plaque burden was visualized by Oil Red O staining of the aortic root. Oil Red O-positive areas were normalized to the size of the vessel wall (Fig. 1a, d). *ApoE*^*−/−*^*LysM*^*wt*^*CB2*^*fl/fl*^ mice treated with JZL184 showed a significant increase in their atherosclerotic plaque burden (39.6 ± 2.1% after JZL184 treatment vs. 32.6 ± 2.4% with DMSO; *n* = 14–15; *p* < 0.05) (Fig. 1a). This effect of JZL184 was blunted in mice lacking the myeloid CB2 receptor (*ApoE*^*−/−*^*LysM*^*cre*^*CB2*^*fl/fl*^), with no significant difference in the atherosclerotic plaque burden between JZL184- and DMSO-treated animals (35.0 ± 1.9%, JZL184 vs. 34.0 ± 2.5%, DMSO; *n* = 14–16; *p* = 0.75).

To detect infiltrating macrophages in the aortic vessel wall, immunohistochemical staining of CD68 was performed (Fig. 1b and in further detail in Supplementary Fig. S1a). Macrophage infiltration was significantly elevated in the cre-negative animals after JZL treatment (JZL184 31.6 ± 1.5% vs DMSO 25.6 ± 2.1%; *n* = 14–15; *p* < 0.05) but not in their cre-positive littermates, lacking the myeloid CB2 receptor (JZL184 29.9 ± 1.2% vs DMSO 27.0 ± 2.1%; *n* = 14–17; *p* = 0.27).

For further characterization of the plaques, neutrophils within the atherosclerotic vessel wall were detected by immunofluorescent staining of Ly6G (Fig. 1c and in further detail in Supplementary Fig. S1b). Treatment with JZL184 showed an increase in neutrophil granulocytes in cre-negative mice compared to DMSO controls (JZL184 8.2 ± 1.2% vs DMSO 4.1 ± 0.7%; *n* = 12–16; *p* < 0.01). Again, this effect was attenuated in cre-positive mice lacking the myeloid CB2 receptor (6.1 ± 0.9%, JZL184 vs. 4.1 ± 0.8%, DMSO; *n* = 13–17; *p* = 0.11).

Collagen deposition was studied using Picrosirius red staining (Supplementary Fig. S1c). Interestingly, treatment with JZL184 of mice expressing the myeloid CB2 receptor resulted in a significantly increased collagen content within the atherosclerotic plaque (DMSO vs. JZL184, 1.10 ± 0.19% vs. 2.75 ± 0.51%; *p* < 0.01; *n* = 11–12). This effect was abolished in mice lacking the myeloid CB2 receptor (DMSO vs. JZL184, 1.55 ± 0.32% vs. 1.41 ± 0.22%; *p* > 0.99; *n* = 14).

### 2-AG-Induced Atherogenesis in ApoE-Deficient Mice Was Unaffected in Mice Lacking Endothelial CB2 Receptor Expression

**Fig. 2:**
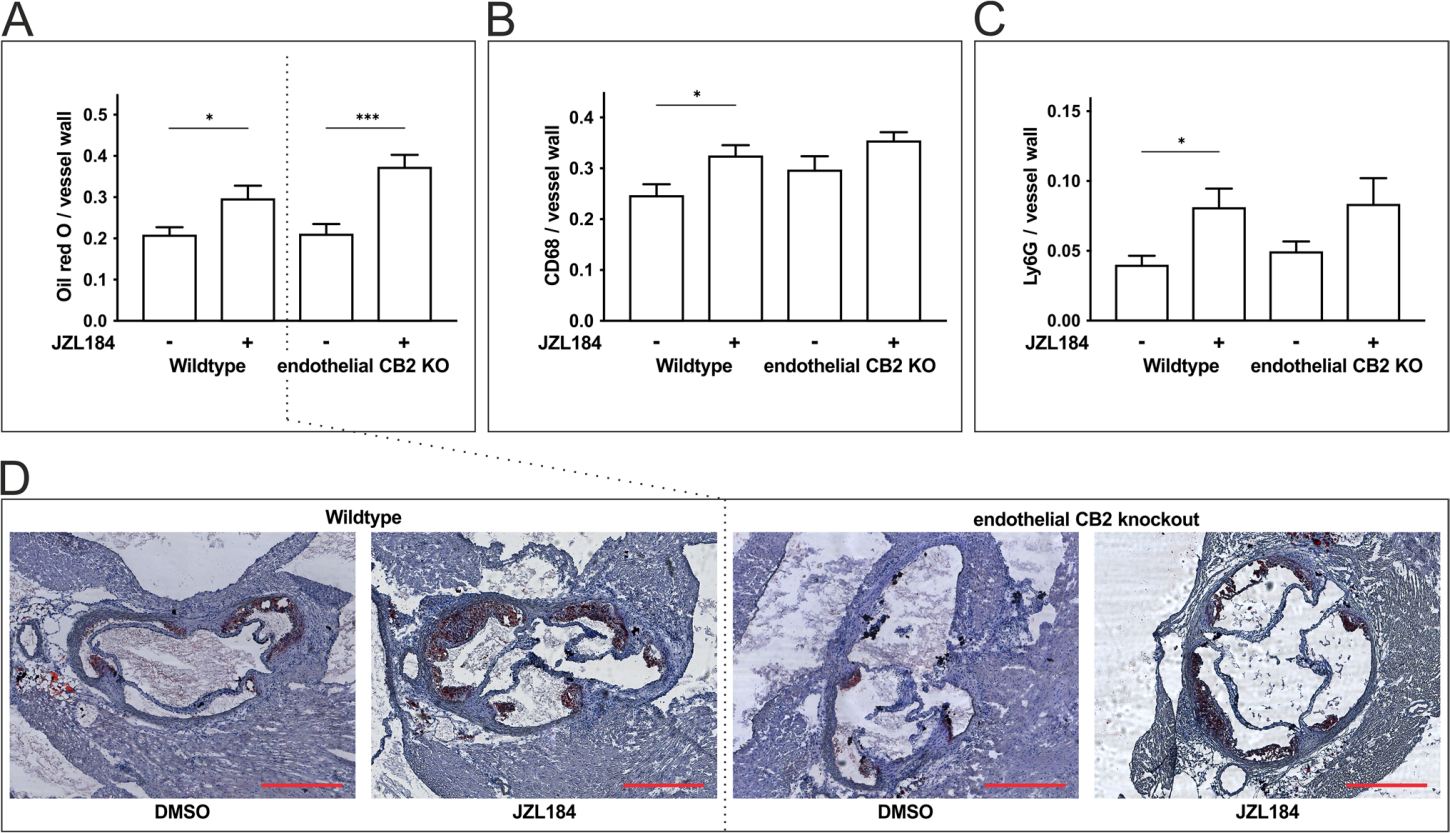
Assessment of atherogenic effects in mice lacking the endothelial CB2 receptor and in wildtype animals after treatment with JZL184. Staining of aortic valve sections in mice with an endothelial-specific knockout of the CB2 receptor and the respective wildtype controls. **a**,** d** Atherosclerotic plaque volume (Oil Red), **b** monocyte/macrophage (CD68), and **c** neutrophil (Ly6G) extravasation in mice with and without the endothelial CB2 receptor expression. Data are presented as the mean ± standard error of the mean; *n* = 9–14; * ≤ 0.05, *** *p* ≤ 0.001, as assessed by Student’s *t* test. Scale bar, 500 μm. DMSO, dimethyl sulfoxide; JZL184, inhibitor of monoacylglycerol lipase

To evaluate the effects of the cell-specific knockout of the CB2 receptor in endothelial cells (*ApoE*^*−/−*^*Tie2*^*cre*^*CB2*^*fl/fl*^) on the development of atherosclerotic plaques, Oil Red O staining of the aortic wall was performed. The treatment of cre-negative animals (*ApoE*^*−/−*^*Tie2*^*wt*^*CB2*^*fl/fl*^) with JZL184 led to a significant increase in the atherosclerotic plaque burden (29.5 ± 3.3% after JZL184 treatment vs. 20.7 ± 2.1% with DMSO; *n* = 14; *p* < 0.05) (Fig. 2a, d). Interestingly, the effect of JZL184 was not attenuated in mice lacking the endothelial CB2 receptor (*ApoE*^*−/−*^*Tie2*^*cre*^*CB2*^*fl/fl*^) compared to DMSO controls (37.1 ± 3.1% after JZL184 treatment vs. 20.9 ± 2.6% with DMSO; *n* = 10–12; *p* < 0.001) (Fig. 2a).

For further characterization of the atherosclerotic plaque morphology, a CD68 staining was performed to identify monocytes and macrophages (Fig. 2b and in further detail in Supplementary Fig. S1d). JZL184 significantly enhanced the accumulation of monocytes and macrophages within the vessel wall (JZL184 32.3 ± 2.2% vs. DMSO 24.5 ± 2.4%; *n* = 13; *p* < 0.05) in cre-negative mice (*ApoE*^*−/−*^*Tie2*^*wt*^*CB2*^*fl/fl*^). This effect was mildly impaired in the cre-positive animals (*ApoE*^*−/−*^*Tie2*^*cre*^*CB2*^*fl/fl*^) lacking the endothelial CB2 receptor (JZL184 35.3 ± 1.8% vs. DMSO 29.5 ± 2.8%; *n* = 9–12; *p* = 0.13).

Finally, Ly6G staining was performed to identify neutrophils infiltrating the vessel wall (Fig. 2c and detailed in Supplementary Fig. S1e). Elevated levels of 2-AG after JZL184 injection led to an increased infiltration of neutrophils in the cre-negative mice (*ApoE*^*−/−*^*Tie2*^*wt*^*CB2*^*fl/fl*^), compared to vehicle-treated mice (JZL184 8.1 ± 1.4% vs DMSO 3.9 ± 0.7%; *n* = 13–14; *p* < 0.05). In mice lacking the endothelial CB2 receptor (*ApoE*^*−/−*^*Tie2*^*cre*^*CB2*^*fl/fl*^), this effect was less pronounced (JZL184 8.3 ± 1.9% vs DMSO 4.8 ± 0.8%; *n* = 10–12; *p* = 0.095).

### Ex Vivo Assessment of Reactive Oxygen Species

In order to gain further mechanistic insights into the effects of 2-AG on atherogenesis, the formation of reactive oxygen species (ROS), as a marker of local inflammation, was measured in mice with/without elevated 2-AG levels in the presence and absence of the CB2 receptor on myeloid cells.

**Fig. 3:**
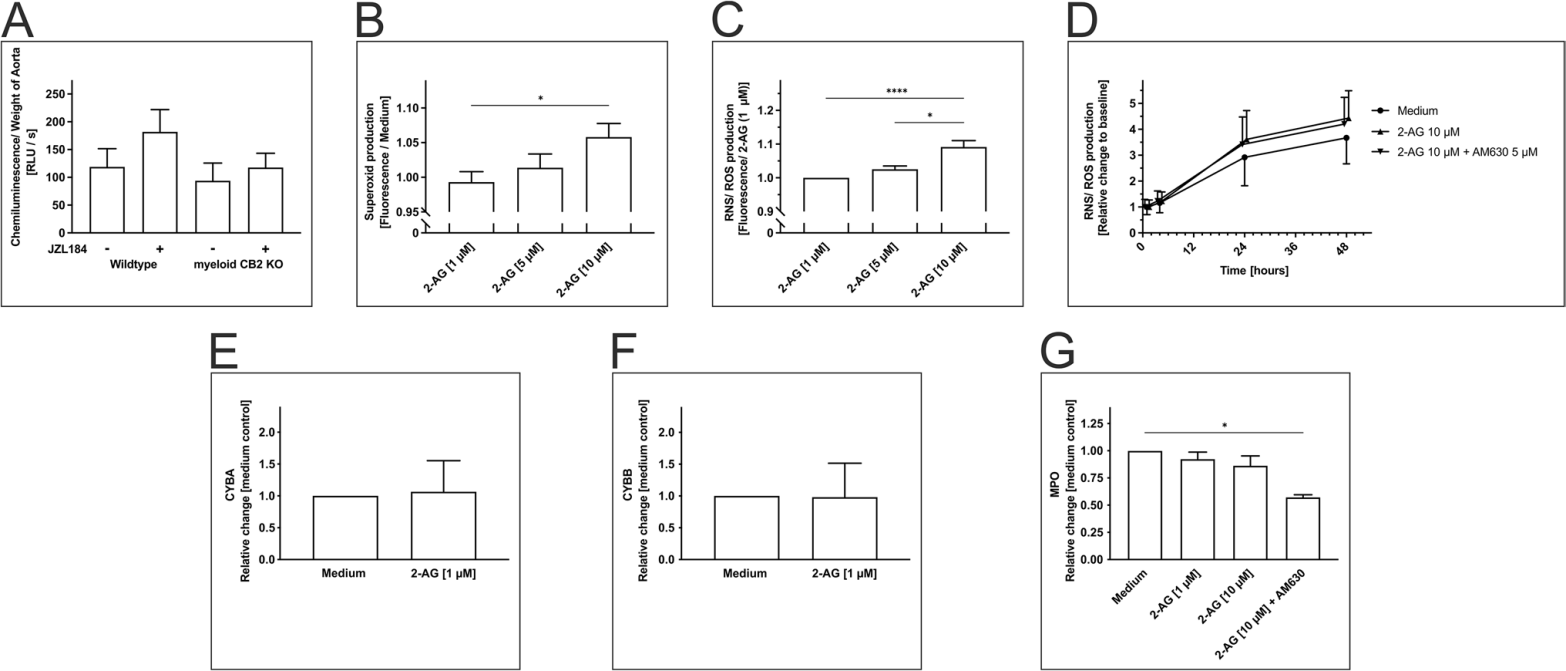
ROS production and quantification of the transcription of the key regulating enzymes CYBA, CYBB, and MPO by 2-AG in THP-1 cells. **a** L-012 chemiluminescence of aortic tissue. **b** Superoxide and **c**,** d** ROS and RNS production by THP-1 cells, **e**,** f** NADPH oxidase 2 (CYBA, CYBB) transcription, and **g** MPO in cell supernatants after stimulation with 2-AG. Data are presented as the mean ± standard error of the mean; *n* ≥ 3; * ≤ 0.05, *** *p* ≤ 0.001, assessed by ANOVA and Bonferroni correction or Kruskal–Wallis test with subsequent Dunn’s multiple comparison for nonparametric testing. 2-AG, 2-arachidonoylglycerol; AM630, selective inhibitor of CB2; CYBA, cytochrome b-245 alpha polypeptide/cytochrome b-245 light chain; CYBB, cytochrome b-245 heavy chain; DMSO, dimethyl sulfoxide; JZL184, inhibitor of monoacylglycerol lipase; MPO, myeloperoxidase; RNS, reactive nitrogen species; ROS, reactive oxygen species

Mice expressing the CB2 receptor (*ApoE*^−/−^*LysM*^wt^*CB2*^fl/fl^) showed numerically higher ROS levels, when treated with JZL184 compared to DMSO-treated controls. However, this effect did not reach statistical significance (JZL184 181.7 ± 40.1 RLU/s vs. DMSO 118.7 ± 32.9 RLU/s; *n* = 15–16; *p* > 0.99) (Fig. 3a). This numerical difference was not observed in mice lacking the myeloid CB2 receptor (*ApoE*^−/−^*LysM*^cre^*CB2*^fl/fl^, JZL184 117.7 ± 25.7 RLU/s vs. DMSO 93.9 ± 32.0 RLU/s; *n* = 15–16; *p* > 0.99) (Fig. 3a).

### 2-AG Increases ROS and RNS Production In Vitro

These differences in ROS production in vivo prompted us to examine the influence of 2-AG on ROS and RNS production in vitro*.* Therefore, we stimulated THP-1 cells with increasing concentrations of 2-AG and measured the ROS/RNS as well as superoxide production. Further, we aimed to determine the effect of CB2 on ROS/RNS production by the co-stimulation of THP-1 cells with 2-AG in combination with AM630, which is an inverse agonist for the CB2 receptor.

Stimulation of THP-1 cells with 2-AG [10 µM] caused a slight, yet significant, dose-dependent increase in superoxide (Fig. 3b) and ROS/RNS production (2-AG [10 µM], 1.09 ± 0.019, *n* = 17, *p* < 0.01; 2-AG [5 µM], 1.025 ± 0.010, *n* = 17, *p* < 0.05) (Fig. 3c).

Co-stimulation with AM630 did not attenuate the effect within 48 h of observation, and no significant differences in ROS/RNS production between 2-AG [10 µM] with or without AM630 could be measured (2-AG [10 µM], 4.43 ± 1.05 vs. 2-AG [10 µM] + AM630, 4.20 ± 1.03; *n* = 13; *p* > 0.99) (Fig. 3d).

### Neither the Release of MPO Nor the Transcription of NADPH Oxidase Seems To Be Responsible for the Increase of ROS and RNS

To further investigate the pathological mechanism behind the 2-AG dependent elevation of ROS/RNS and superoxide, we took a closer look at the generation of these species. Molecular oxygen is converted to superoxide by the NADPH oxidase 2 (NOX-2) or through its subunits cytochrome b-245 light chain (CYPA) and cytochrome b-245 heavy chain (CYBB). After the conversion of superoxide to hydrogen peroxide, it is converted to ROS by myeloperoxidase (MPO). Therefore, we measured CYBA, CYBB, and MPO concentration in the supernatants of THP-1 cells after stimulation with 2-AG.

Stimulation of THP-1 with 2-AG [1 µM] for 1 h did not change the expression of CYBA (2-AG [1 µM] 1.06 ± 0.49, *n* ≥ 3; *p* = 0.90) (Fig. 3e). Similarly, stimulation with 2-AG [1 µM] did not affect the expression of CYBB (0.98 ± 0.53 after 2-AG [1 µM], *n* ≥ 3; *p* = 0.97) (Fig. 3f).

Additionally, MPO in cell supernatants of THP-1 cells was measured 48 h after stimulation with 2-AG [1 µM], 2-AG [10 µM], 2-AG [10 µM] + AM630 [5 µM], or medium. No difference compared to medium control was detected after stimulation with 2-AG (2-AG [1 µM]. 0.96 ± 0.06, *n* = 5, *p* > 0.99; 2-AG [10 µM], 0.87 ± 0.09, *n* = 5; *p* = 0.74) (Fig. 3g). Yet, co-stimulation with AM630 attenuated the basal MPO production (2-AG [10 µM] + AM630 [5 µM], 0.57 ± 0.02, *n* = 3, *p* < 0.05 (Fig. 3g).

### Stimulation with 2-AG Increases the Concentration of IL-1β In Vitro

**Fig. 4:**
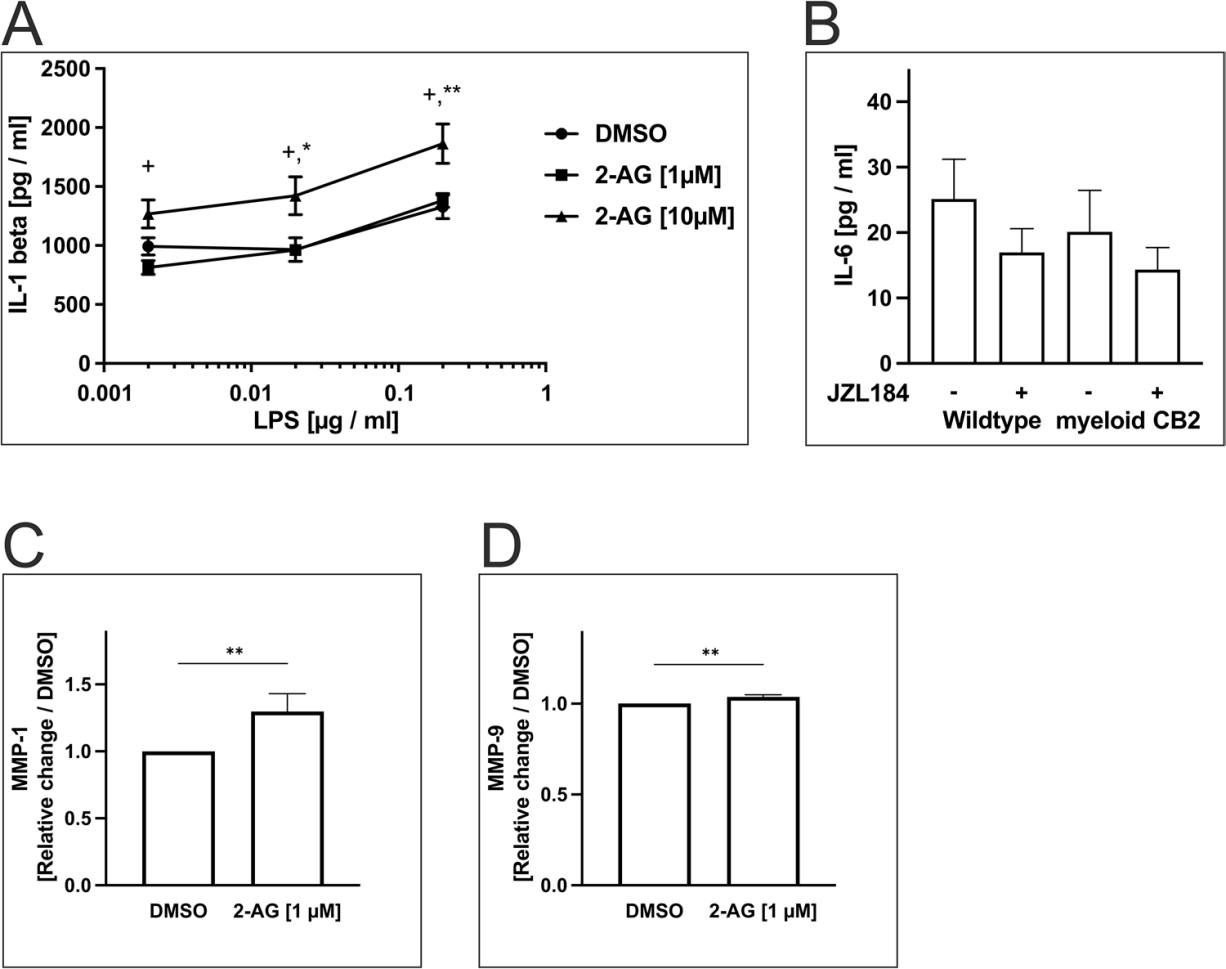
Quantification of 2-AG-induced production of IL-1β in vitro and IL-6 in vivo. **a** IL-1β production by THP-1 cells after co-stimulation with LPS and 2-AG. **b** IL-6 plasma concentration in mice with and without the myeloid CB2 receptor after receiving JZL184 or DMSO treatment. **c** Release of MMP-1 into the supernatants of human monocytes after stimulation with 2-AG. **d** Release of MMP-9 into the supernatants of human monocytes after stimulation with 2-AG. Data are presented as the mean ± standard error of the mean. *n* = 4–12; * ≤ 0.05, ** *p* ≤ 0.01 compared to DMSO; + *p* ≤ 0.05 compared to 2-AG [1 µM], assessed by ANOVA and Bonferroni correction or Kolmogorov–Smirnov test for nonparametric testing; DMSO, dimethyl sulfoxide, IL, interleukin; JZL184, inhibitor of monoacylglycerol lipase; LPS, lipopolysaccharide; MMP, matrix metalloproteinase; THP-1, human monocytic cell line

The IL-1β/IL-6 axis is an important signaling pathway for human and murine atherosclerosis. Earlier studies have shown a link between IL-1β and the ECS for inflammatory diseases other than atherosclerosis as well as neurological diseases [18, 34]. This prompted us to examine whether the IL-1β/IL-6 pathway is involved in mediating endocannabinoid-associated effects in the presented cellular and murine models: IL-1β was measured in supernatants from PBMC after co-stimulation with 2-AG and LPS in various concentrations. We observed a dose-dependent increase in IL-1β release upon stimulation with 2-AG and LPS (2-AG [10 µM], 1864 ± 168.2 pg/ml, *n* = 12, *p* < 0.01; 2-AG [1 µM], 1383 ± 58.9 pg/ml, *n* = 12, *p* < 0.05; DMSO, 1329 ± 101.5 pg/ml) (Fig. 4a).

### Levels of Circulating IL-6 Are Not Altered in Mice Lacking the Myeloid CB2 Receptor After Treatment with JZL184

Given this increase in IL-1β upon stimulation with 2-AG in vitro, we measured the levels of IL-1β and IL-6 in murine plasma samples. However, no differences in IL-6 were detected between the treatment groups (*ApoE*^*−/−*^*LysM*^*cre*^*CB2*^*fl/fl*^, JZL184 14.3 ± 3.4 pg/ml vs. DMSO 20.1 ± 6.4 pg/ml, *n* = 4–6, *p* = 0.41; *ApoE*^*−/−*^*LysM*^*wt*^*CB2*^*fl/fl*^, JZL184 17.0 ± 3.6 pg/ml vs. DMSO 25.1 ± 6.1 pg/ml, *n* = 8–12, *p* = 0.33) (Fig. 4b). The concentration of IL-1β remained below the lowest detection range in all animals.

### Release of MMP-1 and MMP-9 from Monocytes Is Enhanced After Stimulation with 2-AG

After stimulation with 2-AG [1 µM], the release of matrix metalloproteinase (MMP)-1 from monocytes into the supernatant significantly increased compared to DMSO control (2-AG, 1.30 ± 0.133-fold; *n* = 7; *p* < 0.01) (Fig. 4c). Additionally, the concentration of MMP-9 in the supernatant was enhanced compared to DMSO control. This effect was statistically significant, but the relative change appears to be negligible (2-AG, 1.04 ± 0.02-fold; *n* = 10; *p* < 0.01) (Fig. 4d).

## Discussion

The present study aimed to elucidate the influence of 2-AG and its receptor CB2 on atherogenesis. In the early stages of atherosclerosis, an atherogenic environment leads to activation of endothelial cells and increases leukocyte adhesion, while later on, monocytes are guided into the intima by chemokines and sustain continuous inflammation [20]. In the present study, we demonstrated that elevated concentrations of the endocannabinoid 2-AG promote atherogenesis in ApoE-deficient mice, accompanied by a significant increase in macrophages and neutrophils infiltrating the vessel wall. In the applied in vivo model, the effect of 2-AG was mitigated in animals lacking the myeloid CB2 receptor. In those mice, the plaque burden decreased, as did the number of infiltrating immune cells. These findings corroborate previous data by our own group and by others, where 2-AG was also reported to expedite atherogenesis, whereas inhibition of the CB2 receptor abolished this effect [12].

In our present study, pharmacological inhibition of MAGL also resulted in a higher collagen content in murine atherosclerotic plaques. Myeloid-specific depletion of the CB2 receptor abolished this effect. These data agree with previous findings by Vujic et al., where a constitutional knockout of MAGL in ApoE^−/−^ mice (with subsequently elevated 2-AG plasma levels) led to an increased collagen content in atherosclerotic plaques [40].

However, an increased collagen content in atherosclerotic plaques must not be mistaken as a surrogate for an increased plaque stability in rodent models. There are counteracting roles of MMPs, either beneficial by mediating intimal thickening or harmful by thinning the extracellular matrix. Besides, there are several differences in the expression patterns and the pathophysiological role between humans and mice. For example, while MMP-1 is abundantly expressed in human endothelial cells and monocytes and contributes to plaque instability, MMP-1 expression in mice is sparse. However, in one study, artificial expression of human MMP-1 in ApoE^−/−^ mice resulted in smaller atherosclerotic plaques [19, 25]. These data are supported by the present study, where MMP-1 release from human monocytes increased upon stimulation with 2-AG, whereas 2-AG enhanced collagen deposition in WT mice. Therefore, the question whether a higher collagen content translates into increased plaque stability will have to be assessed in future experiments using the appropriate animal models or virtual histology studies in humans.

Interestingly, CB2 receptor deficiency in endothelial cells did not provide any benefit towards the plaque burden during the pharmacological elevation of 2-AG, although levels of infiltrating monocytes and neutrophils were found to be decreased. Thus, myeloid, and not endothelial, expression of the CB2 receptor appears to promote atherogenesis in the context of elevated 2-AG levels.

In an earlier study, we demonstrated that 2-AG promotes endothelial dysfunction and leukocyte recruitment [8]. 2-AG might contribute to atherosclerosis via endothelial cell activation, but in our present study, endothelial CB2 receptor activation does not appear to modulate the atherosclerotic plaque burden. One possible explanation might be that the endothelial damage in the present ApoE knockout mouse model is already so pronounced and that endothelial CB2 activation has little or no further impact on endothelial dysfunction. Meanwhile, CB2 receptor activation appears to be a fundamental mechanism in the activation of myeloid cells in the context of atherogenesis. This might explain why the knockout of endothelial CB2 receptor expression does not change the overall atherosclerotic plaque burden, while the knockout of myeloid CB2 receptor expression does so.

Treatment with JZL184 increased the amount of neutrophils within atherosclerotic plaques compared to DMSO control, even though this effect was not statistically significant in mice lacking the endothelial or myeloid CB2 receptor. While past studies on CB receptor expression on neutrophils were inconclusive, there is growing consensus that neutrophils do not express CB1 and CB2 receptors [4, 37]. However, murine CB2 receptor depletion increased neutrophil recruitment in a murine model of endotoxemia, probably mediated by expanded MMP-9 secretion [15]. Another study demonstrated that 2-AG activates human neutrophils, which promotes the transmigration of additional neutrophils independent of the CB receptors [4]. Altogether, the observed accumulation of neutrophils might be a CB receptor-independent effect.

The CB1 and the CB2 receptors are both subject to autoinduction by their respective ligands [1, 11]. In line with these data, we demonstrated in an earlier study that a decrease in 2-AG results in reduced CB2 receptor expression in vascular tissue of ApoE^−/−^ mice. However, CB1 receptor expression in vascular tissue was marginal in this trial and not dependent on 2-AG levels [11]. On the contrary, another study demonstrated that the CB2 activity upregulates CB1 receptor expression in Jurkat cells [1]. Altogether, evidence about the interplay between CB1 and CB2 receptors in the context of elevated endogenous ligands is scarce [1]. Further research is needed, taking into account the cell-specific expression patterns of the respective receptors.

We and others have reported 2-AG to induce macrophage migration in vitro in a CB2-dependent fashion, which might explain the in vivo effects from our current study [12, 17]. Moreover, the present study introduces two possible triggers for the atherogenic effect of 2-AG that are independent of the CB2 receptor: the production of ROS and the secretion of IL-1β.

It is well-known that ROS production is associated with endothelial dysfunction and inflammation and plays a crucial part in atherogenesis [16, 36]. In addition, several studies provide evidence that the ECS is involved in the modulation of ROS/RNS production [21]. Therefore, we decided to quantify ROS production in murine aortic tissue and in cultured human monocytes. While 2-AG promoted ROS production in vitro, this effect was less pronounced in vivo. In vitro, the inverse CB2 receptor agonist AM630 attenuates basal MPO production, while 2-AG stimulation had no effect.

Several studies have underlined the importance of the IL-1β/IL-6 axis in atherosclerotic disease. For example, the CANTOS trial showed beneficial effects of targeting IL-1β [33]. For IL-6, a distinct homeostasis appears to be necessary to prevent atherogenesis: high levels of IL-6 are clearly connected to atherosclerosis, but on the other hand, atherosclerosis is enhanced in IL-6-deficient mice [6, 36]. The endocannabinoid system influences the expression of IL-6 in several ways. While Chang et al. postulated that 2-AG hampers the expression of IL-6, Saroz et al. showed that activation of the CB2 receptor increases the induction of IL-6 [2, 35]. We measured the levels of IL-6 in mice treated with JZL184. Interestingly, we could not detect any effect on IL-6 levels in JZL184-treated animals.

Meanwhile, our in vitro data suggest that 2-AG activates the NLRP3 inflammasome in a dose-dependent and CB receptor-independent fashion, leading to the liberation of proinflammatory IL-1β. While this is, to our knowledge, the first report to link 2-AG to NLRP3 activation, one previous study has demonstrated that the endocannabinoid *N*-arachidonoylethanolamine (AEA) promotes the excretion of IL-1β in a diabetic mouse model [13].

We subsequently measured IL-1β in plasma samples of atherosclerotic mice, but IL-1β levels remained consistently below the assay’s lower limit of detection. Therefore, the activation of the IL-1β/IL-6 axis by 2-AG is an interesting novel finding in vitro, but it remains unlikely to be a driver of atherosclerosis in our mouse model.

The current study underlines the importance of the endocannabinoid system to the pathophysiology of atherosclerosis in a murine model. Several studies by our lab and by others have also highlighted the importance of the endocannabinoid system to human cardiovascular disease [10, 23, 29, 30, 38]. However, the endocannabinoid system is difficult to use as a drug target, given its ubiquitous expression and its cell- and tissue-specific effects in various pathologies. Thus, cell-specific analyses of ECS effects are warranted to pave the way for the development of targeted therapies. Our present study may represent the first step in this quest.

## Study Limitations

In the present study, we examined cellular and molecular effects that are crucial for the development of atherosclerosis. All data were generated in vivo in mice and in vitro in human models. Although those models are established and well-studied, the mechanisms may still vary in humans.

## Conclusions

Taken together, data from the present study demonstrate that 2-AG promotes atherogenesis, whereas the absence of the myeloid CB2 receptor attenuates this effect. On the other hand, the endothelial CB2 receptor appears not to be involved in this mechanism. Three potential atherogenic effects of 2-AG were substantiated by our in vitro experiments: the increase in macrophage migration, the production of ROS, and the activation of the NLRP3 inflammasome. Meanwhile, the pathophysiological relevance of these effects in human atherosclerosis remains to be elucidated.

## Supplementary Information

Supplementary Figure 1 CD68-, Ly6G- and picrosirius red staining of the aortic valve sections. Staining of aortic valve sections in mice with either myeloid-specific knockout of the CB2 receptor **(a**, **b**, **c)** or endothelial-specific knockout of the CB2 receptor **(d**, **e)** and the respective wildtype controls. Monocyte/macrophage (CD68; **a**, **d**) and neutrophil (Ly6G; **b**, **e**) extravasation in mice with and without myeloid or endothelial CB2-receptor expression was measured. Collagen deposition (white asterisks) within atherosclerotic plaques (white arrows) of mice with a myeloid-specific knockout of the CB2 receptor and the respective wildtype controls was studied using picrosirus red staining (**c**). For better visualization, bar charts from Fig. 1b,c and Fig. 2 b,c are presented on the left side. Data are presented as the mean ± standard error of the mean; n 9 - 17; * ≤ 0.05, ** p ≤ 0.01, as assessed by student’s t-test. Scale bar, 500 μm. CB2, Cannabinoid receptor 2; DMSO, dimethyl sulfoxide; JZL184, inhibitor of monoacylglycerol lipase. (PNG 2989 kb)

High resolution image (TIF 14919 kb)

Supplementary Figure 2 Graphical summary of the experimental plan. ApoE, Apolioprotein E; CB2, Cannabinoid receptor 2; CD68, Cluster of Differentiation 68; DMSO, dimethyl sulfoxide; JZL184, inhibitor of monoacylglycerol lipase; LC-MRM, Liquid chromatography-multiple reaction monitoring; Ly6G, Lymphocyte antigen 6 complex locus G6D; ROS, reactive oxygen species; (PNG 677 kb)

High resolution image (TIFF 8127 kb)

ESM 1 Supplementary Table S1 TagMan® probes used for qPCR. This table list all TagMan® probes used in this study for performing real-time PCR analysis. Supplementary Table S2 2-AG concentrations in Plasma and Aortic tissue. All mice were treated with either 5 mg/kg body weight of JZL184 or the vehicle (PBS/Kolliphor/DMSO) for four weeks. The final injection of JZL184 or vehicle was given four hours before sacrifice. 2-arachidonoylglycerol levels were quantified by liquid chromatography-multiple reaction monitoring. Data are presented as mean ± standard error of the mean; p-values as indicated, assessed by student’s t-test. DMSO, dimethyl sulfoxide; fl, flanked by loxP; JZL184, inhibitor of monoacylglycerol lipase; WT, wildtype. Supplementary Table S3 Endocannabinoid plasma levels. ApoE^-/-^- with either an endothelial CB2-Knockout, a myeloid CB2-Knockout or their wt littermates received JZL184 (5 mg/kg body weight) or the vehicle (PBS/Kolliphor/DMSO) for four weeks. Plasma was collected four hours after the last infection and endogenous levels of the eCB were assessed using liquid chromatography-multiple reaction monitoring. Data are presented as mean ± standard error of the mean; p-values as indicated, assessed by student’s t-test. DMSO, dimethyl sulfoxide; eCB, endocannabinoids; fl, flanked by loxP; JZL184, inhibitor of monoacylglycerol lipase; WT, wildtype. Supplementary Table S4 Clinical Parameters. Clinicial parameters were measured prior to sacrifice. Body weight, heart rate and systolic blood pressure did not neither differ between the different genotype nor between the treatment strategies. Data are presented as mean ± standard error of the mean; p-values as indicated, assessed by student’s t-test. DMSO, dimethyl sulfoxide; fl, flanked by loxP; JZL184, inhibitor of monoacylglycerol lipase; WT, wildtype. (DOCX 35 kb)

## Abbreviations

2-AG: 2-Arachidonoylglycerol
eCB: Endocannabinoids
CB2: Cannabinoid receptor 2
JZL184: Inhibitor of monoacylglycerol lipase
MAGL: Monoacylglycerol lipase
MMP: Matrix metalloproteinase
MPO: Myeloperoxidase
ROS: Reactive oxygen species
RNS: Reactive nitrogen species
NOX: NADPH oxidase
NOX2: NADPH oxidase 2 (p91-PHOX/cytochrome b (-245) heavy chain)
CYBB: Cytochrome b-245 heavy chain
CYBA: Cytochrome b-245 alpha polypeptide/cytochrome b-245 light chain (p22-PHOX)
IL-6: Interleukin 6
IL-1β: Interleukin 1β
