# Supplementary figures and images for: Myeloid But Not Endothelial Expression of the CB2 Receptor Promotes Atherogenesis in the Context of Elevated Levels of the Endocannabinoid 2-Arachidonoylglycerol
Source: J Cardiovasc Transl Res. 2022 Sep 30;16(2):491–501. doi: 10.1007/s12265-022-10323-z (PMC10151305; doi:10.1007/s12265-022-10323-z)

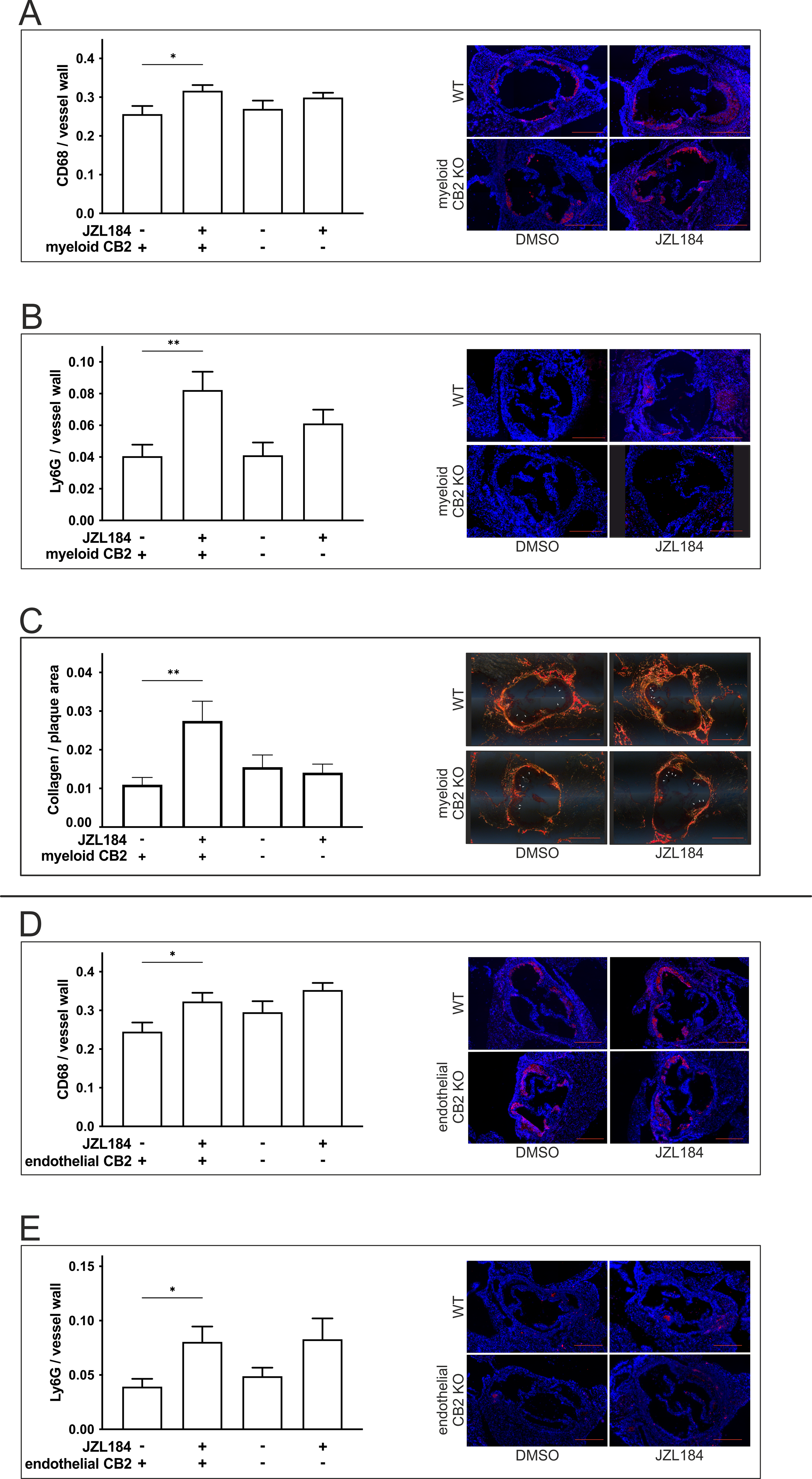

Supplement: Supplementary file 1 — CD68-, Ly6G- and picrosirius red staining of the aortic valve sections. Staining of aortic valve sections in mice with either myeloid-specific knockout of the CB2 receptor (a, b, c) or endothelial-specific knockout of the CB2 receptor (d, e) and the respective wildtype controls. Monocyte/macrophage (CD68; a, d) and neutrophil (Ly6G; b, e) extravasation in mice with and without myeloid or endothelial CB2-receptor expression was measured. Collagen deposition (white asterisks) within atherosclerotic plaques (white arrows) of mice with a myeloid-specific knockout of the CB2 receptor and the respective wildtype controls was studied using picrosirus red staining (c). For better visualization, bar charts from Fig. 1b,c and Fig. 2 b,c are presented on the left side. Data are presented as the mean ± standard error of the mean; n 9 - 17; * ≤ 0.05, ** p ≤ 0.01, as assessed by student’s t-test. Scale bar, 500 μm. CB2, Cannabinoid receptor 2; DMSO, dimethyl sulfoxide; JZL184, inhibitor of monoacylglycerol lipase. (PNG 2989 kb) [file 12265_2022_10323_Fig5_ESM.png]

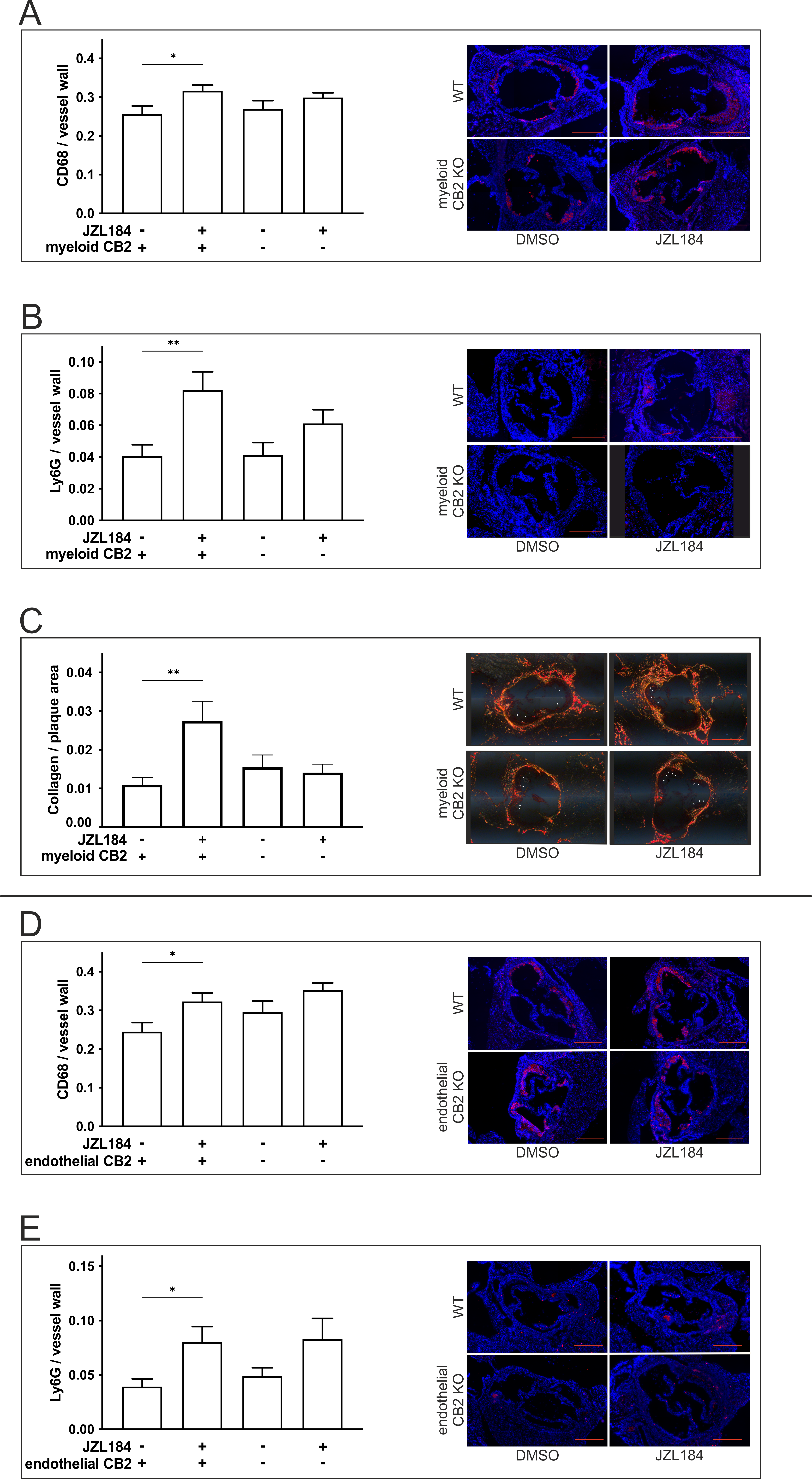

Supplement: Supplementary file 2 — High resolution image (TIF 14919 kb) [file 12265_2022_10323_MOESM1_ESM.tif]

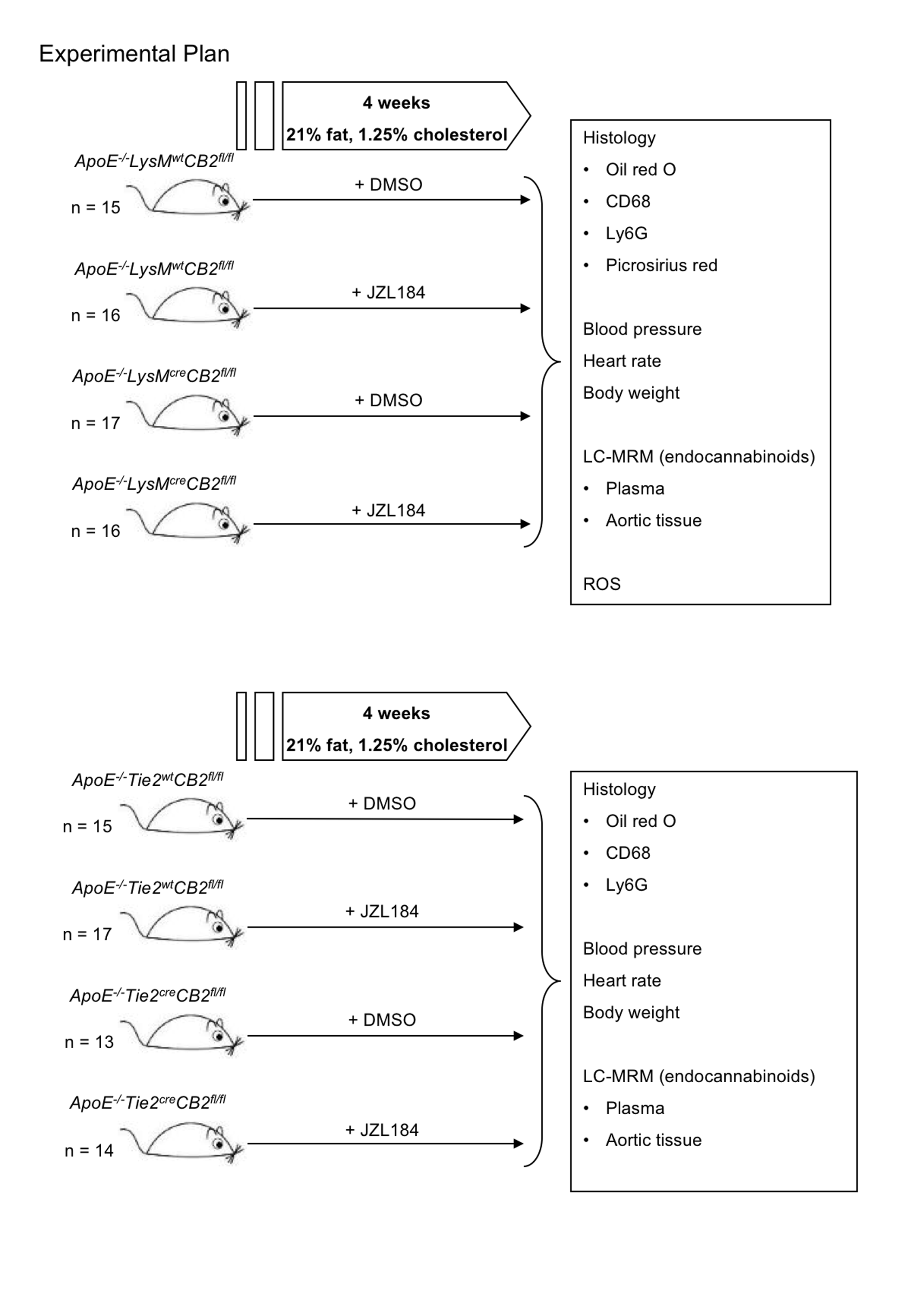

Supplement: Supplementary file 3 — Graphical summary of the experimental plan. ApoE, Apolioprotein E; CB2, Cannabinoid receptor 2; CD68, Cluster of Differentiation 68; DMSO, dimethyl sulfoxide; JZL184, inhibitor of monoacylglycerol lipase; LC-MRM, Liquid chromatography-multiple reaction monitoring; Ly6G, Lymphocyte antigen 6 complex locus G6D; ROS, reactive oxygen species; (PNG 677 kb) [file 12265_2022_10323_Fig6_ESM.png]

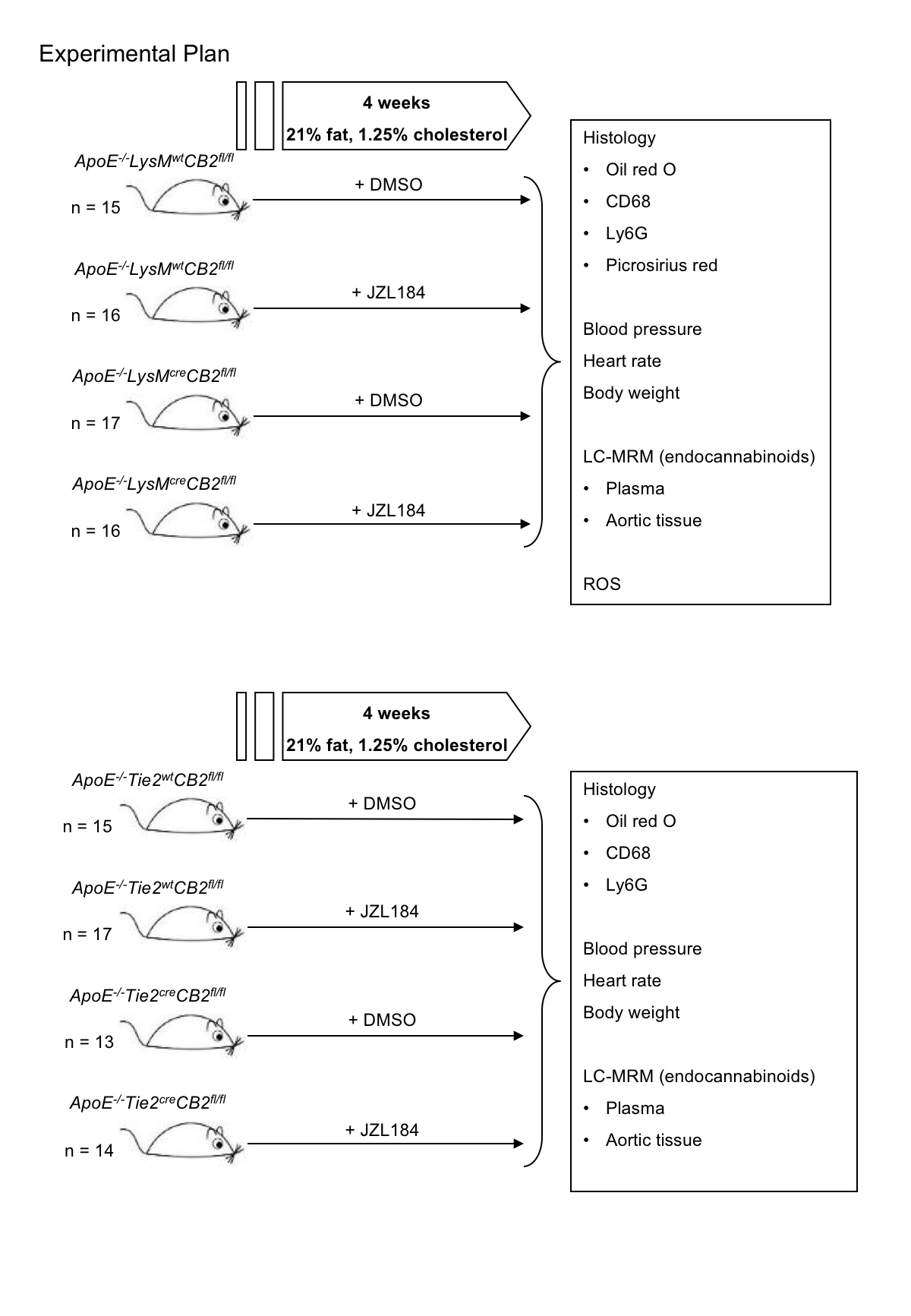

Supplement: Supplementary file 4 — High resolution image (TIFF 8127 kb) [file 12265_2022_10323_MOESM2_ESM.tiff]
