# Supplementary material for: Myeloid But Not Endothelial Expression of the CB2 Receptor Promotes Atherogenesis in the Context of Elevated Levels of the Endocannabinoid 2-Arachidonoylglycerol
Source: J Cardiovasc Transl Res. 2022 Sep 30;16(2):491–501. doi: 10.1007/s12265-022-10323-z (PMC10151305; doi:10.1007/s12265-022-10323-z)
Supplement: Supplementary file 5 — Supplementary Table S1 TagMan® probes used for qPCR. This table list all TagMan® probes used in this study for performing real-time PCR analysis. Supplementary Table S2 2-AG concentrations in Plasma and Aortic tissue. All mice were treated with either 5 mg/kg body weight of JZL184 or the vehicle (PBS/Kolliphor/DMSO) for four weeks. The final injection of JZL184 or vehicle was given four hours before sacrifice. 2-arachidonoylglycerol levels were quantified by liquid chromatography-multiple reaction monitoring. Data are presented as mean ± standard error of the mean; p-values as indicated, assessed by student’s t-test. DMSO, dimethyl sulfoxide; fl, flanked by loxP; JZL184, inhibitor of monoacylglycerol lipase; WT, wildtype. Supplementary Table S3 Endocannabinoid plasma levels. ApoE-/-- with either an endothelial CB2-Knockout, a myeloid CB2-Knockout or their wt littermates received JZL184 (5 mg/kg body weight) or the vehicle (PBS/Kolliphor/DMSO) for four weeks. Plasma was collected four hours after the last infection and endogenous levels of the eCB were assessed using liquid chromatography-multiple reaction monitoring. Data are presented as mean ± standard error of the mean; p-values as indicated, assessed by student’s t-test. DMSO, dimethyl sulfoxide; eCB, endocannabinoids; fl, flanked by loxP; JZL184, inhibitor of monoacylglycerol lipase; WT, wildtype. Supplementary Table S4 Clinical Parameters. Clinicial parameters were measured prior to sacrifice. Body weight, heart rate and systolic blood pressure did not neither differ between the different genotype nor between the treatment strategies. Data are presented as mean ± standard error of the mean; p-values as indicated, assessed by student’s t-test. DMSO, dimethyl sulfoxide; fl, flanked by loxP; JZL184, inhibitor of monoacylglycerol lipase; WT, wildtype. (DOCX 35 kb) [file 12265_2022_10323_MOESM3_ESM.docx]

**Supporting information**

**Supplementary Tables:**

**S1 Table: TaqMan® probes used for qPCR.**

| **TaqMan® Gene Expression Assay ID** | **Target Gene** | **Vendor** | **Cat Num** |
| --- | --- | --- | --- |
| Hs01071088_m1 | NOX1  NADPH oxidase 1 | ThermoFisher Scientific | 4331182 |
| Hs03044361_m1 | CYBA  Cytochrome b-245 alpha chain | ThermoFisher Scientific | 4331182 |
| Hs00166163_m1 | CYBB  Cytochrome b-245 beta chain | ThermoFisher Scientific | 4331182 |
| Hs01379108_m1 | NOX4  NADPH oxidase 4 | ThermoFisher Scientific | 4331182 |
| Hs00225846_m1 | NOX5  NADPH oxidase. EF-hand calcium binding domain 5 | ThermoFisher Scientific | 4331182 |

**S2 Table: 2-AG concentrations in Plasma and Aortic tissue**

| **Myeloid Knockout** | **ApoE^-/-^LysM^cre^CB2^fl/fl^** | | | | **ApoE^-/-^LysM^wt^CB2^fl/fl^** | | | |
| --- | --- | --- | --- | --- | --- | --- | --- | --- |
|  | DMSO | JZL184 | p | n | DMSO | JZL184 | p | n |
| **Plasma [pmol/ml]** | 16.9 ± 2.2 | 59.4 ± 7.0 | < 0.0001 | 16-17 | 18.5 ± 3.0 | 58.0 ± 5.2 | < 0.0001 | 15-16 |
| **Aortic tissue [nmol/g]** | 42.4 ± 9.1 | 110.1 ± 27.2 | < 0.05 | 3-4 | 38.4 ± 6.8 | 135.5 ± 32.5 | < 0.05 | 4 |
|  |  |  |  |  |  |  |  |  |
| **Endothelial Knockout** | **ApoE^-/-^Tie2^cre^CB2^fl/fl^** | | | | **ApoE^-/-^Tie2^wt^CB2^fl/fl^** | | | |
|  | DMSO | JZL184 | p | n | DMSO | JZL184 | p | n |
| **Plasma [pmol/ml]** | 33.5 ± 2.9 | 73.1 ± 6.4 | < 0.0001 | 13-14 | 42.7 ± 3.3 | 71.4 ± 5.1 | < 0.0001 | 14-17 |
| **Aortic tissue [nmol/g]** | 61.0 ± 14.1 | 239.5 ± 36.2 | < 0.001 | 13-14 | 68.9 + 13.2 | 173.2 ± 20.0 | < 0.001 | 15-17 |

**S3 Table: Endocannabinoid plasma levels**

| **Myeloid Knockout** | | **N-arachidonoylethanolamide** | | **Arachidonic acid** | | | **Palmitoylethanolamide** | | |
| --- | --- | --- | --- | --- | --- | --- | --- | --- | --- |
|  | **n** | **[pmol/ml]** | **p** | **[nmol/ml]** | | **p** | **[pmol/ml]** | | **p** |
| ApoE^-/-^LysM^wt^CB2^fl/fl^ + DMSO | 15 | 0.70 ± 0.04 | 0.036 | 1.65 ± 0.13 | | 0.634 | 63.72 ± 7.24 | | 0.055 |
| ApoE^-/-^LysM^wt^CB2^fl/fl^ + JZL184 | 16 | 0.82 ± 0.03 |  | 1.55 ± 0.14 | |  | 47.71 ± 3.78 | |  |
| ApoE^-/-^LysM^cre^CB2^fl/fl^ + DMSO | 17 | 0.73 ± 0.09 | 0.376 | 1.74 ± 0.29 | | 0.542 | 47.92 ± 2.88 | | 0.281 |
| ApoE^-/-^LysM^cre^CB2^fl/fl^ + JZL184 | 16 | 0.82 ± 0.06 |  | 1.54 ± 0.11 | |  | 43.54 ± 2.77 | |  |
|  | |  | | |  | | |  | |
| **Endothelial Knockout** | | **N-arachidonoylethanolamide** | | **Arachidonic acid** | | | **Palmitoylethanolamide** | | |
|  | **n** | **[pmol/ml]** | **P** | **[nmol/ml]** | | **p** | **[pmol/ml]** | | **p** |
| ApoE^-/-^Tie2^wt^CB2^fl/fl^ + DMSO | 15 | 0.99 ± 0.06 | 0.584 | 2.72 ± 0.51 | | 0.146 | 49.54 ± 4.14 | | 0.061 |
| ApoE^-/-^Tie2^wt^CB2^fl/fl^ + JZL184 | 17 | 1.04 ± 0.07 |  | 1.87 ± 0.30 | |  | 59.22 ± 2.91 | |  |
| ApoE^-/-^Tie2^cre^CB2^fl/fl^ + DMSO | 13 | 1.08 ± 0.06 | 0.835 | 2.43 ± 0.24 | | 0.073 | 50.93 ± 2.80 | | 0.253 |
| ApoE^-/-^Tie2^cre^CB2^fl/fl^ + JZL184 | 14 | 1.10 ± 0.07 |  | 1.93 ± 0.13 | |  | 56.64 ± 3.91 | |  |

**S4 Table: Clinical Parameters**

| **Myeloid Knockout** | **Heart Rate** | | | **Systolic blood pressure** | | | **Body weight** | | |
| --- | --- | --- | --- | --- | --- | --- | --- | --- | --- |
|  | **[1/min]** | **n** | **p** | **[mmHg]** | **n** | **p** | **[g]** | **n** | **p** |
| ApoE^-/-^LysM^wt^CB2^fl/fl^ + DMSO | 672 ± 38 | 8 | 0.470 | 126 ± 7 | 8 | 0.749 | 26.0 ± 0.7 | 15 | 0.514 |
| ApoE^-/-^LysM^wt^CB2^fl/fl^ + JZL184 | 629 ± 44 | 8 |  | 123 ± 8 | 8 |  | 25.3 ± 0.8 | 16 |  |
| ApoE^-/-^LysM^cre^CB2^fl/fl^ + DMSO | 631 ± 42 | 8 | 0.930 | 135 ± 7 | 8 | 0.128 | 25.2 ± 0.8 | 17 | 0.402 |
| ApoE^-/-^LysM^cre^CB2^fl/fl^ + JZL184 | 637 ± 51 | 8 |  | 115 ± 9 | 8 |  | 26.3 ± 1.0 | 16 |  |
|  |  | | |  | | |  | | |
| **Endothelial Knockout** | **Heart Rate** | | | **Systolic blood pressure** | | | **Body weight** | | |
|  | **[1/min]** | **n** | **p** | **[mmHg]** | **n** | **p** | **[g]** | **n** | **p** |
| ApoE^-/-^Tie2^wt^CB2^fl/fl^ + DMSO | 672 ± 27 | 8 | 0.397 | 151 ± 7 | 8 | 0.627 | 25.3 ± 1.1 | 15 | 0.339 |
| ApoE^-/-^Tie2^wt^CB2^fl/fl^ + JZL184 | 700 ± 16 | 8 |  | 146 ± 7 | 8 |  | 26.6 ± 0.9 | 17 |  |
| ApoE^-/-^Tie2^cre^CB2^fl/fl^ + DMSO | 685± 18 | 8 | 0.586 | 148 ± 7 | 8 | 0.778 | 25.8 ± 1.1 | 13 | 0.842 |
| ApoE^-/-^Tie2^cre^CB2^fl/fl^ + JZL184 | 703 ± 26 | 8 |  | 145 ± 8 | 8 |  | 25.5 ± 0.8 | 14 |  |
